# Comparison of different feedback modalities for the training of procedural skills in Oral and maxillofacial surgery: a blinded, randomized and controlled study

**DOI:** 10.1186/s12909-020-02222-1

**Published:** 2020-09-24

**Authors:** Lukas B. Seifert, Carlos Herrera-Vizcaino, Philipp Herguth, Jasmina Sterz, Robert Sader

**Affiliations:** 1 Department of Oral, Cranio-Maxillofacial, and Facial Plastic Surgery, Goethe University Frankfurt, Theodor-Stern-Kai 7, 60590 Frankfurt, Germany; 2 Department of Trauma, Reconstructive and Hand Surgery, Goethe University Frankfurt, Theodor-Stern-Kai 7, 60590 Frankfurt, Germany

**Keywords:** Procedural skills, Maxillofacial surgery, Feedback, Undergraduate education, Dental students

## Abstract

**Background:**

The feedback given to students plays an important role in their efficiency related to learning practical skills. In the present study, diverse feedback modalities have been investigated. Our hypothesis is that individualized and unsupervised video feedback can produce a similar learning experience as performing practical skills in an oral and maxillofacial surgery setting with conventional direct expert feedback (control group).

**Methods:**

This prospective, randomized, controlled, and blinded study compared direct expert feedback (DEF), individualized video feedback (IVF) and unsupervised video feedback (UVF). The participants were fourth-year dental students from University Goethe in Frankfurt. The students were assigned to one of the three feedback methods (*n* = 20 per group) using simple randomization. All participants watched an instruction video for an interdental (‘Ernst’) ligature and periphery venous catheterization. Next, the students were video recorded performing the tasks by themselves (pre-test). Following this, every student received feedback using one of the above-mentioned feedback modalities. The participants then performed the same task again while being video recorded (post-test) to measure the acquired competence. Six weeks later, the students participated in an objective structured clinical examination (OSCE) to evaluate their long-term knowledge retention. All examiners were blinded regarding the students’ instructional approach and their affiliation in terms of the learning group.

**Results:**

For the interdental ligature, we found significant improvements in performance in each feedback modality group between the pre-test and post-test (*p* < 0.001). UVF had the strongest effect on performance time. The comparison between each group in the post-test showed no significant differences between the three groups.

**Conclusion:**

This study showed that IVF and UVF can be considered an alternative or adjunct to conventional methods (i.e. DEF) when learning procedural skills in oral and maxillofacial surgery. However, DEF showed to be the most effective method of feedback and therefore preferable in teaching.

## Background

One of the biggest challenges in both medical and dental training is to provide students with the skills needed for their future work. In daily practice, a wide range of psychosocial to practical-technical skills must be mastered at a very high level [1].Especially against the background of a continuously growing number of multimorbid patients in the dental practice, the knowledge and capacity to carry out basic medical procedures for emergency treatment will be of great relevance in the future [2, 3]. The knowledge of how to perform the placement of a peripheral venous catheter to apply emergency medication in such scenarios is required to initiate early emergency treatment. Another frequent complication in the dental practice is an accidental mandibular fracture during the extraction of third molars [4]. Knowledge of how to perform an interdental ligation with wires can help to stabilize the fracture segments and reduce the patient’s pain in these situations [5]. However, previous studies have shown that dental students are insufficiently prepared for the application of practical and theoretical skills when treating dental and medical emergencies [6]. Possible reasons for these findings are that the teaching of these skills is not sufficiently represented in the dental curricula [6]. Furthermore, the students report receiving little feedback when learning these skills in order to develop a learning effect in the long-term [6, 7].

An accepted definition of feedback in medical education is “specific information between a trainee’s observed performance and a standard, given with the intent to improve the trainee’s performance” [7]. Due to this, feedback plays a crucial role when teaching practical skills and, in many cases, it determines the learning success of a trainee [8]. Even though medical educators frequently believe that they give feedback to their medical trainees, the trainees report that feedback is rare [9, 10]. One possible reason for this is that the students often don’t recognize that they get feedback as it is not structured and well-planned [11].

Due to this, the effectiveness of the diverse structured modalities of given feedback has been investigated in medical education. Recently, media-supported forms of feedback have been used extensively as an effective modality to enhance feedback [12]. Particularly, the use of video recordings to provide effective feedback has been evaluated as a valuable resource in medical education. In a previous study by Xeroulis et al., computer-based video feedback was found to significantly improve the learners’ technical skills in suturing and knot tying [13]. In another study by Farquharson et al., similar results were obtained by comparing verbal feedback to verbal feedback coupled with video feedback [12].

For dental education however, the use of media-supported forms of feedback has not been investigated sufficiently. Therefore the aim of the present study is to investigate the effectiveness of various media-supported feedback forms when teaching procedural skills (peripheral venous catheter and interdental wire-ligation) in the discipline of oral and maxillofacial surgery (OMS). Our hypothesis is that media-supported video feedback can produce a similar learning outcome when performing practical skills as the conventional and often used direct expert feedback method.

## Methods

### Study design

All of the ethical principles for medical research involving human subjects dictated by the 1975 declaration of Helsinki, as revised in 2013, were considered. Concordant with the Ethics Board at the University Medical School, no ethical permission was necessary to conduct the study. The study was prospective, blinded, randomized, and controlled with the following parallel arms (feedback methods):

Control group: Direct expert feedback (DEF).

Intervention group 1: Individualized video feedback (IVF).

Intervention group 2: Unsupervised video feedback (UVF).

The study measured the student’s improvement of performance and the time taken to perform a task directly after an introduction exercise (T0) and a second exercise after receiving feedback (T1). Finally, to measure the long-term learning retention, a final examination (T2) was performed six weeks after the post-test.

The assignment of students to one of the learning groups per training week with a maximum of six students per group who passed through the teaching units together occurred prior to the training week, independent of the authors and independent of study participation by the deanery. The allocation of the learning groups in the study to the three instructional approaches was performed alternately.

### Study participants and study conduction

**Fig. 1:**
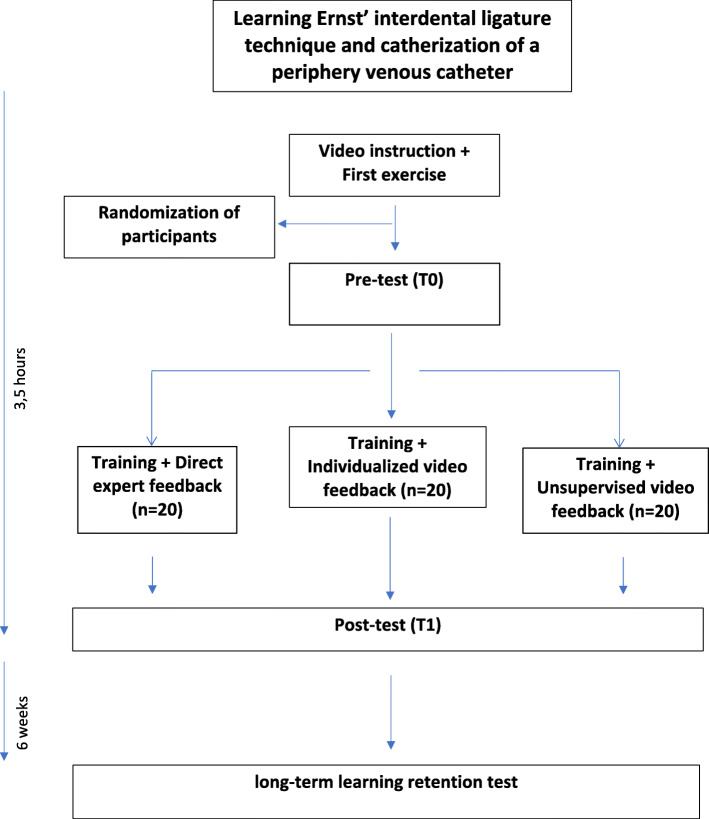
Diagram that represents the participant flow in the study and the timeline

The study participants were fourth-year dentistry students from the University Goethe of Frankfurt in the period of 2018–2019 attending a compulsory internship which includes a five-day rotation through every section of the Department of Oral, Cranio-Maxillofacial and Facial Plastic Surgery, i.e. the operating room, the outpatient clinic and the emergency department. Before starting their rotation, students have to complete practical skills training which has been described in greater detail in a previous publication [14].Teaching was held in small groups ranging from four to six students by the same instructor (a 4th year resident who is responsible for the undergraduate education of the Department) throughout the course of the study. Prior to the study beginning, the instructor received training which included the learning objectives of the practical skills training, a tutor manual that included an explanation on how to correctly perform each skill as well as a timetable and blueprint and trained on the use of the five-step feedback sheet to give the students structured expert feedback. Sixty students signed an informed consent of participation after receiving an explanation of the study process and objectives from which they could withdraw at any time (Fig. 1). They were instructed not to conduct additional training activities during the course of the study.

#### Pre-test evaluation

### Practical skills training and measurements of pre-test evaluation

**Fig. 2:**
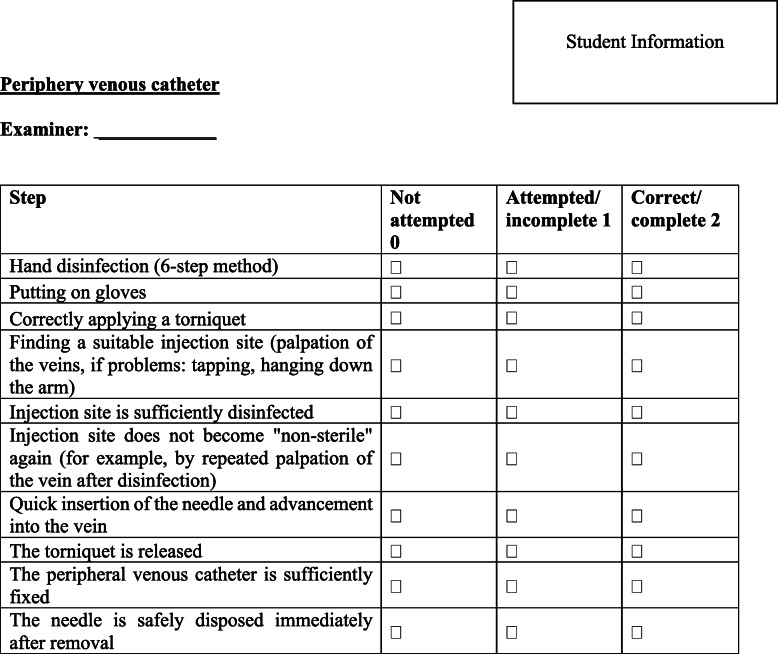
Global rating scale used for the performance measurement of the periphery venous catheter

**Fig. 3:**
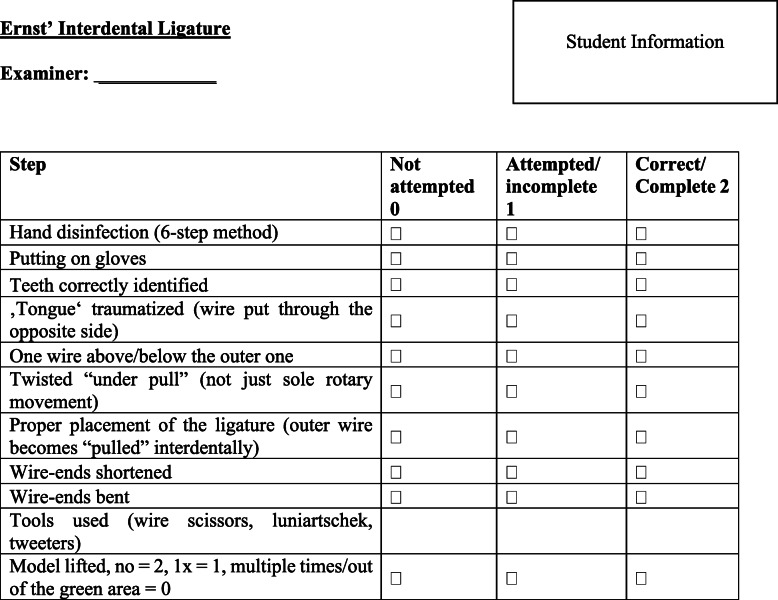
Global rating scale used for the performance measurement of the Ernst’ interdental ligature

In the practical skills training, an emergency situation was simulated in which the students had to insert a periphery venous catheter. In the first exercise, it evaluated the correct use of gloves, the placement of a tourniquet, their knowledge of periphery-venous anatomy, preparing a sterile working surface, placing the catheter and the fixation of the catheter (Fig. 2). The students performed the exercise on a phantom injection arm (Gaumard Scientific, USA). The second exercise involved the first aid treatment of a mandibular fracture using an interdental ligature technique (‘Ernst’ ligature). In this exercise, the correct identification of the fracture line, the placement of the ligature, cutting and twisting the endings of the wire and checking the stability of the ligature were evaluated (Fig. 3). The students performed the exercise on a patient simulator (KaVo Dental GmbH, Biberach, Germany). Before taking part in the practical skills training, all of the students received instructions through a standardized teaching video for each skill. The videos included step-by-step instructions in real-time with comments, hints and frequent mistakes that should be avoided in the performance of the aforementioned skills. The process was based on the tutor’s manual and global rating scale.

### Feedback methods

After receiving the instructions, the students performed the skills by themselves for 30 min. Subsequently, the students were video recorded while performing each skill one last time as a performance measurement that was evaluated by the examiners (T0). This step was followed by the students individually receiving one of the feedback methods investigated in this study (T1). The time of the execution of the practical skills was also documented at T0 and T1.

#### Direct expert feedback (control group)

In this group, the students were supervised by the instructor while performing the skills. During the 30 min of practice, the instructor observed each student one at a time performing the task at least once. This was followed by giving each student individual feedback using a five-step feedback sheet. The five steps in the feedback protocol assessed what went well, what could be improved, what went badly, what was missing, and what the take-home message was for each student. Immediately after the feedback, the students practiced again for 30 min before repeating the exercise while again being video recorded for the subsequent assessment (T1).

#### Individualized video feedback

In this group, feedback was given by the instructor using the same five step-feedback sheet after watching each student performance. The feedback sessions lasted for 30 min. Immediately after the feedback, the students practiced again for 30 min before repeating the exercise while again being video recorded for a later assessment (T1).

#### Unsupervised video feedback

As feedback, the students received once again the standardized video instructions and they were instructed to give themselves feedback using the same five step-feedback sheets. The feedback sessions were performed individually by each student and lasted for 30 min. Immediately after the feedback, the students practiced again for 30 min before repeating the exercise while again being video recorded for later assessment (T1).

#### Long-term retention

To measure long-term retention, an objective structured clinical examination (OSCE) format focused on OMS (OMS-OSCE) took place 6 weeks later (T2). ^1514^ During this time interval, the students did not perform any further exercises or receive any feedback. A regular OSCE is composed of eight 5-min stations, with four of them verifying theoretical skills and four of them assessing practical skills. The practical stations assessed the task “catheterization of a periphery venous catheter” and the second station evaluated the task “interdental ligature”, as described above. Again, the students were video recorded for later assessment (T2).

### Performance measurement

The evaluation of their performance was done using a previously validated global rating scale (GRS; Figures 2 and 3) [15]. This consists of a trinary scoring scale (0 points for not done, 1 point for done but incorrect, and 2 points for done and correct) based on the checklist used in the tutor’s manual.^1514^By adding the aforementioned points, an average performance score was obtained. The global rating scales implemented were primarily piloted in previous undergraduate trainings and afterwards validated by two independent, blinded examiners. In addition, the content validity was ensured as part of an expert workshop with didactic and surgical experts as well as through its repeated application and adaption in the context of the previous studies [15–17] and OSCE exams. For the present study, two examiners received an educational course as calibration and to gain experience using the GRS. The inter-rater reliability was measured using Cohen’s kappa coefficient (﻿κ = 0.84). The performance of the acquired competences in relation to both skills of the study was measured during the practical skills training (T0), directly after the intervention (T1) and 6 weeks later (T2). The examiners rated the student performance using video recordings of the student’s performance at each point in time (Camera System: Panasonic HC-X929, Osaka, Japan). All examiners had the opportunity to examine the videos only once and they were blinded toward the students’ instructional approach and their study group.

### Statistical analysis

Microsoft Office 2016 (Microsoft Office 2007,© Microsoft Corporation, Redmond, USA) for Mac and SPSS Statistics version 19 (IBM, Armonk, USA) were used for the statistical analysis. The data collected was tested for normal Gaussian distribution using the Shapiro-Wilk normality test. The data from their performance was analyzed using two-way analysis of variance (ANOVA) with a Tukey multiple comparisons test done for all pairs. Time was analyzed using an unpaired two-tailed t-test (α = 0.05, 95% CI of diff.). Cohen’s *d* was used as an additional control test to support the interpretation of the data. A larger absolute value indicates a stronger effect. The results have been presented as the mean and standard deviation (SD), depicted in tables. Statistical significance was considered if *p* < 0.05.

### Sample size estimation

Based on the prior examination results from the years before the intervention and our null hypothesis that alternative feedback methods (IVF and UVF) are not inferior (no-inferiority study) when providing effective feedback compared to traditional methods (DEF), we estimated an average student performance of 70% with a standard deviation of 10% in the OSCE. With an average student number of 65 per semester, a sample size of 56 was calculated based on the following parameters: average student performance = 70%, alpha = 0.05, beta = 0.2 and power = 0.8.

## Results

Fifty-nine students completed the study. There was one drop out in the “Direct expert feedback” group for personal reasons. Every student successfully completed the practical skills training and the implementation of each feedback method within the curricular structure of the training was possible without any complication. All of the results from the pre-test (T0), post-test (T1) and long-term retention test (T2) are shown in Tables 1, 2, 3,4.

**Table 1:** Evaluation of the placement of the interdental ligature at pre-test, post-test and OSCE

	pre-test (T0)	post-test (T1)	OSCE (T2)	*p*-value	Effect size (pre to post)	Effect size (post to OSCE)	Effect size (pre to OSCE)
DEF	13.07 +/− 2.60	16.40 +/− 1.55	16.67 +/− 3.58	*p* < 0.001	1.54	0.09	1.15
IVF	14.04 +/− 1.52	16.26 +/− 1.76	15.83 +/− 2.92	*p* < 0.001	1.35	0.17	0.77
UVF	14.30 +/− 1.69	16.19 +/− 1.50	16.80 +/− 2.46	*p* < 0.001	1.12	−0.29	1.14
*p-*value	*p* = 0.15	*p* = 0.84	*p* = 0.33

**Table 2:** Evaluation of the placement of the periphery venous catheter at pre-test, post-test and OSCE

	pre-test (T0)	post-test (T1)	OSCE (T2)	*p*-value	Effect size (pre to post)	Effect size (post to OSCE)	Effect size (pre to OSCE)
DEF	13.60 +/− 2.67	20.13 +/− 2.03	17.33 +/− 1.54	*p* < 0.001	2.75	−1.55	1.71
IVF	14.09 +/− 2.20	18.45 +/− 1.68	16.59 +/− 2.20	*p* < 0.001	2.22	−0.95	1.13
UVF	15.90 +/− 2.34	18.80 +/− 1.99	16.35 +/− 2.16	*p* = 0.018	1.34	−1.18	0.2
*p*-value	*p* = 0.08	*p* = 0.79	*p* = 0.43

**Table 3:** Time required to perform the procedures at pre-test and post-test

		Time needed pre-test (T0) (min)	Time needed post-test (T1) (min)	Average improvement in speed (min)	Number of students that improved speed (%)	Number of students that didn’t improve speed (%)	p-value
Interdental ligature	DEF	04:19	04:15	0:04 ± 1:13	47%	53%	*p =* 0.56
	IVF	03:42	03:40	0:01 ± 0:36	52%	48%	*p =* 0.75
	UVF	04:07	03:37	0:30 ± 0:42	81%	19%	*p* = 0.02
*p*-value		*p* = 0.15	*p* = 0.08				
Periphery venous catheter	DEF	4:17	3:16	1:00 ± 0:41	100%	0%	*p* = 0.0001
	IVF	3:58	3:17	0:40 ± 0:58	73%	27%	*p* = 0.06
	UVF	4:40	3:26	1:14 ± 1:01	95%	5%	*p* = 0.0004
*p*-value		*p* = 0.16	p = 0.70

**Table 4:** Number of students that improved performance (%)

		Pre-test (T0) Vs Post-test (T1)	Post-test (T1) Vs OSCE (T2)	Pre-test (T0) Vs OSCE (T2)
Interdental ligature	DEF	95%	47%	74%
	IVF	85%	45%	75%
	UVF	80%	60%	85%
Periphery venous catheter	DEF	89%	6%	85%
	IVF	95%	19%	81%
	UVF	85%	15%	50%

### Evaluation of performance - interdental ligature

The intragroup comparison at T0 showed there to be no significant differences between the groups. During the study, all groups improved their performance significantly from T0 to T1 (*p* < 0.001). Additionally, no significant differences between groups were recorded at T1 (*p* = 0.84). The DEF group showed the biggest effect size regarding the improvement from T0 to T1. Notably, the time needed to execute the exercises significantly improved in the UVF group (*p* < 0.02) (Table 3). The intergroup comparison at T0 and T1 did not show there to be any significant differences between the average times of the three groups (*p* = 0.15 und *p* = 0.08 respectively). Comparing the results of performance from T1 with the T2, none of the groups showed any significant differences. The same was observed in the intragroup comparison at T2 where no significant differences (*p* = 0.33) (Cohen’s d from post-test to OSCE: dDEF = 0.09; dIVF = 0.17; dUVF = − 0.29) were found. The biggest improvement was observed by comparing the results from T0 and T2. At T2, significant increases in the overall average score of all groups compared to T0 (*p* < 0.014) were recorded and the highest effect size according to Cohen’s *d* was found in the DEF group (dDEF = 1.15; dIVF = 0.77; dUVF = 1.14) (Table 1).

### Evaluation of performance - periphery venous catheter

The intergroup comparison at T0 showed there to be no significant differences between the groups. Furthermore, the intergroup comparison showed no significant differences between the three groups at T1 (*p* = 0.79). However, the results showed a significant performance improvement in all groups from T0 to T1 (*p* < 0.001). The highest effect size according to Cohen’s *d* was found in the DEF group (dDEF = 2.75; dIVF = 2.22; dUVF = 1.34). The time required to execute the exercises also significantly improved in the DEF Group (*p* = 0.0001) and UVF Group (*p* = 0.0004). The intergroup comparison at T0 and T1 did not show any significant differences between the average times of the three groups (*p* = 0.16 und *p* = 0.70 respectively) (Table 3).

Notably, in the IVF and UVF groups, there was a statistically significant deterioration in performance (*p* = 0.047, *p* = 0.042, respectively) from T1 to the T2. The intragroup comparison at T2 showed there to be no significant differences between the groups (*p* = 0.42) (Cohen’s *d* from post-test to OMS-OSCE: dDEF = − 1.55; dIVF = − 0.95; dUVF = − 1.18). The results at T2 did not diminish to the point of being as low as the results at T0. By comparing the results at T0 and at T2, the analyses showed there to be a significant increase in the average of the overall scores in the DEF Group and IVF Group (*p* = 0.018; *p* = 0.012, respectively). Contrary to the DEF and IVF groups, there were no significant improvements between T1 and T2 in the UVF group (*p* > 0.9). The highest effect size according to Cohen’s *d* was in the DEF Group (dDEF = 1.71; dIVF = 1.13; dUVF = 0.2) (Table 2).

## Discussion

The aim of this single-blinded study was to prospectively investigate the teaching efficacy of three feedback methods: direct expert feedback (control group), individualized video feedback (intervention group 1) and unsupervised video feedback (intervention group 2) in relation to the short- and long-term acquisition of two basic surgical skills. Another aim of this study was to investigate the curricular (‘in vivo’) feasibility of the media-supported feedback methods. Overall, our results revealed significant performance increases for all feedback forms between the pre-test (T0) and the post-test (T1). Furthermore, the re-examination 6 weeks later (T2) revealed good long-term learning retention of the acquired practical skills, especially for the DEF group. The DEF group showed the strongest effect size in the intergroup comparison between all testing ties. However, the intragroup comparison showed that IVF and UVF were not inferior to traditional direct feedback in terms of the mediation of the assessed skills. The implementation of media-supported feedback forms in a curricular setting is completely feasible within the given timeframe of practical skills training week [18].

The correct placement of a peripheral venous catheter, as well as the performance of an interdental ‘Ernst’ ligation, represents two fundamental OMF skills. Because of the rising number of multimorbid patients in the dental practice, the knowledge and capacity to carry out these basic medical procedures for emergency treatment is of great relevance to future dentists [2, 3]. Moreover, the skills examined in this study were selected because they had never been performed before by the 4th year students in contrast to other skills. Previous knowledge, i.e. placing a local anesthesia, would have probably biased our study results since the students already had different levels of knowledge of how to perform these tasks. Furthermore, since the sample size was small, Cohen’s *d* was used as an additional control test to support the interpretation of the data. Cohen’s *d* is defined as the difference between two means divided by the standard deviation of the data, resulting in a unitless value that helps to interpret the effect size of observed results, hence the statistical power of a study. For most types of effect size, a larger absolute value indicates a stronger effect. Furthermore, it can be used as an additional control test since prior studies have shown that significant test results alone are.

not sufficient enough to interpret the data and draw conclusions [19].

Selecting an appropriate method to provide feedback is of paramount importance as the previous studies have demonstrated that feedback determines learning success. An example of this was published by Schüler et al. (2018). The feedback that was provided through practical clinical courses fostered the development of technical, management and communication skills significantly more with large effect sizes compared to the same course without feedback (Schüler et al. 2018). Furthermore, Olms et al. were able to demonstrate that dental students believe individual feedback to be helpful (Olms et al. 2017). According to this, feedback in dental education is found to positively influence the dental students’ autonomous motivation (Orsini 2017) and it also has benefits in terms of the students’ attitude toward the course and their confidence in diagnosis and treatment planning (Lipp 2016).

In the present study, the measurements were registered at three time-intervals, which led to a better classification and understanding of the learning process. In the literature, the action of systematically practicing with the aim of improving performance while receiving feedback is also known as deliberate practice [20]. In this sense, the conducted study could be considered an action of deliberate practice that led to improvement. In addition, the time needed to perform the tasks during T0 and T1 were also registered and a reduction of the time to taken perform was taken as another indicator of improvement. The results of our study show that independently of the feedback method, a high number of students reduced the time taken to perform the task of placing a periphery venous catheter. Contrary to this in the interdental ligature group, a reduction of the time taken to perform in a high number of students was only observed in the UVF group. Learning how to place an interdental ligature appears to require more practice. The UVF feedback method seems to have a higher effect when learning this skill, which makes it more feasible to include in the dental curricula. The reduction of the time taken to perform the task is of relevance, especially since the skills evaluated are to be used in emergency treatments [12, 21]. Taken together, the data in this study adds to the growing body of evidence that suggests that in order to improve through deliberate practice, the student needs to set a task to improve and to define a measurable metric of performance as a guiding point (*for example, a* standardized teaching video) but not necessarily the presence of direct expert feedback. Nevertheless, further research needs to be conducted in this regard.

The study questioned the statement that IVF and UVF lead to a similar improvement in the performance and learning of procedural skills as with traditional DEF. A previous study aimed to evaluate the effect of IVF and UVF on improvements in suturing. The participants were video recorded while suturing and they were scored by two experts during the task. After receiving feedback, the participants were requested to repeat the task. All forms of feedback led to a significant improvement in suturing [22]. In an additional single-blinded study, the candidates received a live demonstration of an intravenous cannulation by an expert. Later on, they were randomized to perform the task in isolation while being recorded. The participants were randomized to receive either DEF or UVF and they were evaluated at 3 time-intervals for 7 weeks. The results showed an improvement in both groups without any significant differences [23]. Similar to our study, the results of the aforementioned studies show that all modalities of feedback investigated were useful regarding the improvement of procedural skills. In our study, a significant performance increase was observed at T1 and T2 compared to T0 for the interdental ligature task. However, with an effect size of 2.75 and 1.54, the DEF group profited more from the training/feedback session than the IVF and UVF groups. A rationale for this could be the presence of the expert during the feedback session. The expert’s know-how allows for the spotting of mistakes more quickly and feedback being given directly. On the contrary, in the IVF and UVF cases, the lack of experience of the students impedes them from recognizing the mistakes made in their own video-recorded performance. Previous studies have also investigated the use of DEF and the effect that it has on the learning progress. The effect of ‘computer-based video instruction’, ‘summary feedback’ and ‘direct feedback’ were compared when teaching suturing and knot-tying skills to medical students. All of the participants received an instructional video and were pre-tested directly after. Every participant obtained a training session utilizing one of the feedback forms. After one month, the student’s long-term retention was tested. ‘Computer-based video instruction’, ‘summary feedback’ and ‘direct feedback’ were all found to be effective based on the acquisition of basic surgical skills [13]. Nevertheless, during the direct feedback, feedback and performance occur concurrently, which requires multitasking. This could be considered a disadvantage compared to the IVF and UVF groups. In the present study, the group with the lower effect was the UVF group. It can be assumed that with only the video, the lack of experience of the students and without the expert’s tips, the students could not identify if any mistakes were made easily. A supplementary video with “frequent mistakes / tips and tricks” could enhance this modality of feedback and it can be used to focus on the specific triggering of errors.

In the present study, the authors decided not to integrate a control group without any feedback. This could be considered a limitation of this study because one might argue that the improvement of the study groups was due to repetition of the task rather than the feedback method used to improve learning. On the other hand, the effectiveness of any type of feedback in comparison to no feedback is already proven in different studies in the field of medical and dental education [24–26]. Due to this, the students included in a control group without feedback would have been disadvantaged. As this is not permitted in a curricular setting ending with a summative assessment as in the setting in which the present study took place, the authors made the conscious decision not to create this kind of control group.

The control measurements at T0 for all three groups showed similar results concerning the practical knowledge in the examined exercises, pointing to a good comparability between the groups at baseline. Furthermore, compared to T0, the results at T2 showed a significant improvement in all groups. Nevertheless, as this was not a real time scenario, the results should be appraised carefully and not directly extrapolated to real life scenarios. Here, the examination itself has to be considered even though it was carried out in a formative way. It has a positive influence on the performance of the students [27]. Further studies should focus on the long-term retention in a real time scenario, which was not possible here due to the curricular setting that the study took part in. On the other hand, the curricular setting is one of the big advantages that the present study has. As a whole semester could be included in the study, we were able to avoid selection bias and to demonstrate that the kinds of feedback used are feasible to integrate into an existing curricular course.

In the present study, the authors were able to demonstrate that UVF is quantitatively equal at improving basic surgical skills to DEF and IVF. This is even more notable as UVF needs no presence of the tutor during the feedback itself.

## Conclusions

Direct expert feedback showed to be the most effective method of feedback and therefore preferable in teaching. However, this study has shown that individualized video feedback and unsupervised video feedback are both acceptable approaches in the acquisition of basic surgical skills. Individualized video feedback and unsupervised video feedback can be considered as useful adjunct to conventional feedback methods when learning procedural skills in oral and maxillofacial surgery.

## Data Availability

The datasets used and/or analyzed during the current study are available from the corresponding author on reasonable request.

## Abbreviations

DEF: Direct expert feedback
IVF: Individualized video feedback
OMS: Oral and Maxillofacial surgery
OSCE: Objective structured clinical examination
UVF: Unsupervised video feedback
